# Chondroitin sulfate disaccharides modified the structure and function of the murine gut microbiome under healthy and stressed conditions

**DOI:** 10.1038/s41598-017-05860-6

**Published:** 2017-07-28

**Authors:** Fang Liu, Na Zhang, Zhaojie Li, Xiong Wang, Hongjie Shi, Changhu Xue, Robert W. Li, Qingjuan Tang

**Affiliations:** 1 College of Food Science and Engineering, Ocean University of China, Qingdao, 266003 China; 2 United States Department of Agriculture, Agriculture Research Service (USDA-ARS), Animal Genomics and Improvement Laboratory, Beltsville, MD 20705 USA

## Abstract

Chondroitin sulfate (CS) has been widely used for medical and nutraceutical purposes due to its roles in maintaining tissue structural integrity. We investigated if CS disaccharides may act as a bioactive compound and modulate gut microbial composition in mice. Our data show that CS disaccharides supplementation for 16 days significantly reduced blood LPS in the mice experiencing exhaustive exercise stress. CS disaccharides partially restored total fecal short-chain fatty acids from the level significantly repressed in mice under the stress. Our findings demonstrated that CS was likely butyrogenic and resulted in a significant increase in fecal butyrate concentration. CS disaccharides had a profound impact on gut microbial composition, affecting the abundance of 13.6% and 7.3% Operational Taxonomic Units in fecal microbial communities in healthy and stressed mice, respectively. CS disaccharides reduced the prevalence of inflammatory Proteobacteria. Together, our findings demonstrated that CS may ameliorate stress-induced intestinal inflammation. Furthermore, CS significantly increased intestinal *Bacteroides acidifaciens* population, indirectly exerting its immunomodulatory effect on the intestine. CS disaccharides had a significant impact on a broad range of biological pathways under stressed condition, such as ABC transporters, two-component systems, and carbohydrate metabolism. Our results will facilitate the development of CS as a bioactive nutraceutical.

## Introduction

Chondroitin sulfate (CS) belongs to a class of sulfated glycosaminoglycans (GAG) that consist of up to hundreds of repeating disaccharide units. The basic disaccharide unit is composed of glucuronic acid (GlcA) and *N*-acetylgalactosamine (GalNAc) with sulfate residues at various positions. Composition heterogeneity of CS is determined by degree of polymerization and sulfation modification. Proteoglycans containing CS polysaccharide chains are ubiquitous and are located in connective tissue matrix, cell surface and basement membranes, or in intracellular granules of certain cells^1^. The CS biosynthesis in humans is very complicated and catalyzed by dozens of enzymes encoded by genes located in at least 14 chromosomes^2^. Gene knockout studies suggest that CS plays a critical role in development and homeostasis of organs and tissues^3^. For example, the data from the mice lacking N-acetygalactosaminyltransferase I, a key enzyme in CS biosynthesis, demonstrate that this gene is necessary for endochondral ossification^3^; and the knockout mice recover more completely from spinal cord injury than wild type mice^4^. Moreover, CS has been proven to possess numerous biological functions. CS serves as extracellular signaling molecules as well as co-receptors or signal modulators^2^. Intriguingly, the sulfation patterns of CS chains have importantly functional implications. The ratio of 6-*O*-sulfation and 4-*O*-sulfation changes drastically during brain development^5^. Furthermore, CS type E (CS-E) with di-sulfate on the GalNAc residue, derived from squid cartilage, exhibits potent antiviral activity and may serve as a receptor for herpes simplex virus^6^. In addition, CS has been suggested as a receptor in placental malaria^7^. Recently, syndecan-1 has been identified as the proteoglycan to which CS-A (mono-sulfation at 4-*O* position) is attached for the recognition of a parasite protein^8^.

As an essential part of structural proteoglycans, such as aggrecan and neurocan, CS is a major component of cartilage and plays a critical role in maintaining tissue structural integrity. As a result, CS has been widely used for its potential medical and nutraceutical properties^9^. CS has been suggested to be responsible for the biological effects of GC protein-derived macrophage activating factor^10^. Recently, glucosamine has been promoted as a dietary supplement for osteoarthritis, likely due to its potential as one of CS precursors. However, its efficacy is still debated^11, 12^.

CS does not appear to be degradable in the tissue or in luminal contents of stomach and small intestine^13^. Poor absorption across small intestine results in a low bioavailability. However, CS can be readily metabolized to component disaccharides in the hindgut, suggesting a role by the gut microbiome^13^. Indeed, *Bacteroides thetaiotaomicron*, one of the major constitutes of the gut microbiome, can rapidly activate the transcription of CS utilization genes after a sudden exposure to CS and then dynamically adjust their transcription according to the rates at which CS is broken down^14^. Furthermore, various *Bacteroides* species possess species-specific dynamics responses to CS availability and to the composition of the bacterial community when CS is the sole carbon source, enabling the coexistence of various species using a given nutrient^15^. The biochemical processes that lead to microbial breakdown of CS have been known. CS is first broken into unsaturated, sulfated disaccharides. The component disaccharides are then desulfated. The desulfated disaccharides are finally hydrolyzed by a β-glucuronidase to produce monosaccharides. In addition to *Bacteroides*, some novel CS-degrading species, such as *Clostridium hathewayi*, have been identified recently^16, 17^. The β-glucuronidase activities can be detected in a broad range of bacteria, including those from the most predominant phyla in the gut microbiome, such as Firmicutes, Bacteroidetes, Proteobacteria, and Actinobacteria, suggesting that many CS disaccharides-degrading bacteria have yet to be discovered.

In the modern society, people constantly endure severe pressure from work and life style changes and ensuing fatigue. As a result, headaches, insomnia, muscle pain, endocrine dyscrasia, and gastrointestinal (GI) disturbance are common manifestations of people under severe stress. Stress and fatigue are associated with intestinal inflammation and oxidative processes^18^. Recently, stress-induced fatigue has been linked to alterations in the gut microbiome^19^. In this study, we attempted to understand potential prebiotic effect of CS disaccharides and their role in modulating the structure and function of the gut microbiome under healthy and exhaustive exercise-induced stressed conditions using a murine model.

## Results

### CS disaccharides may enhance intestinal absorption and promote kidney function

**Figure 1:**
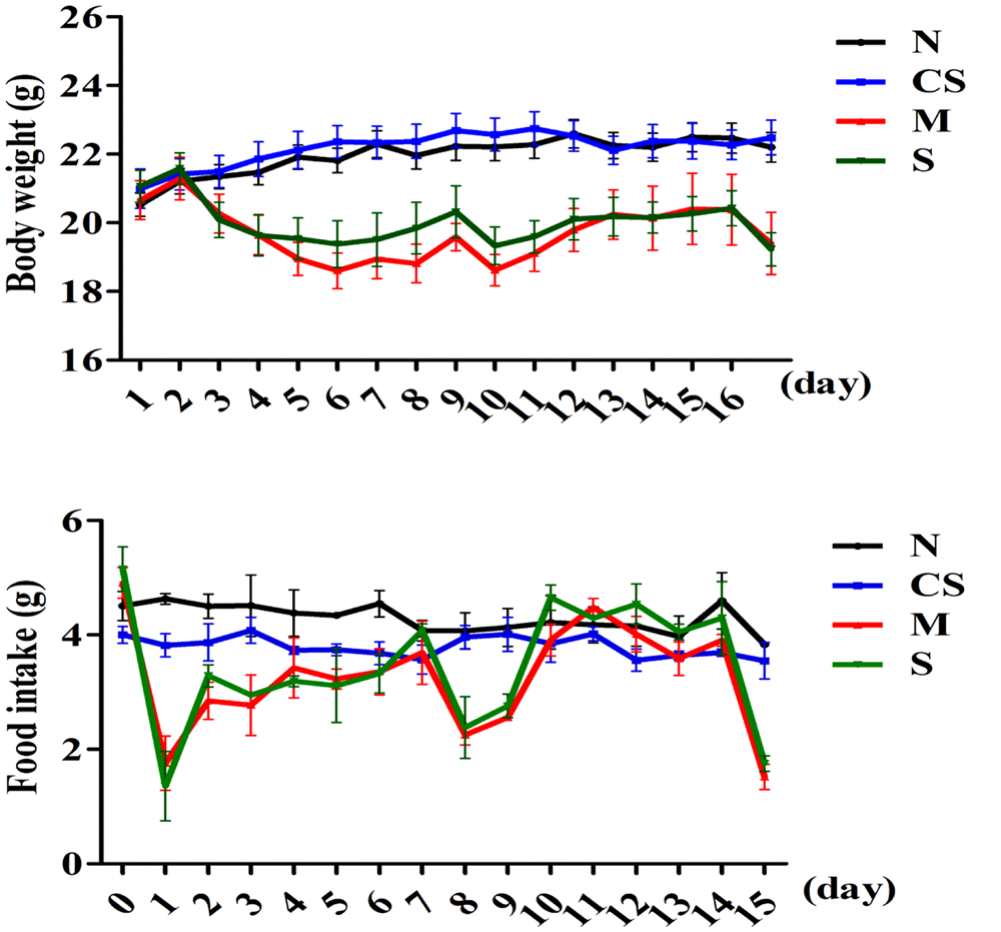
The changes in bodyweight and feed intake during the 16-day experimental period. N: healthy mice + PBS; CS: healthy mice supplemented with a daily dose of 150 mg/kg bodyweight of CS disaccharides for 16 days; M: mice subjected to exhaustive exercise stress + PBS; S: the stressed mice supplemented with a daily dose of 150 mg/kg bodyweight of CS disaccharides for 16 days.

**Figure 2:**
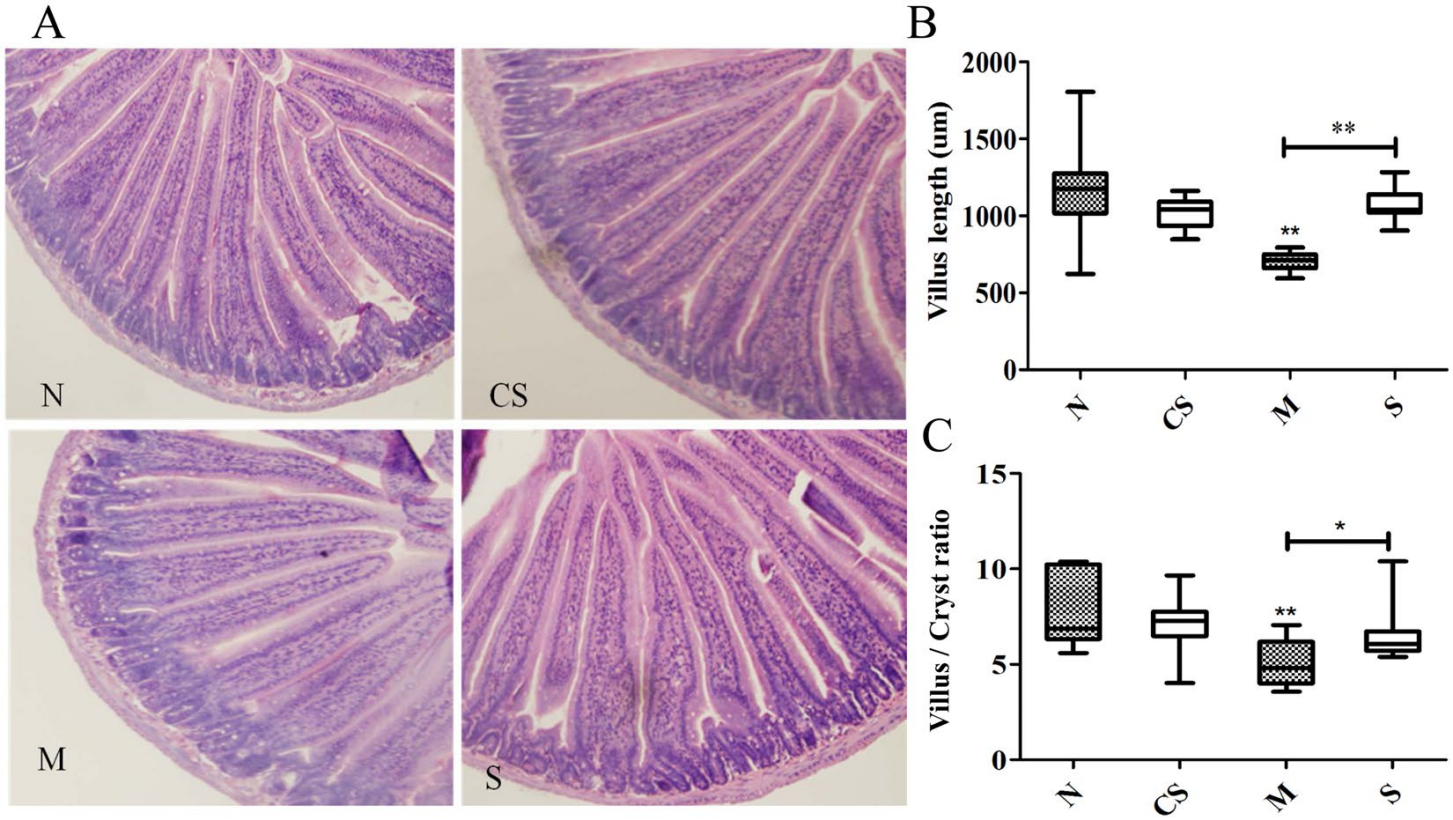
Intestinal morphology and structure of the mice after the 16-day experimental period. (**A**) Ileum morphology; (**B**) Villus length; (**C**) Villus/Cryst ratio. N: healthy mice + PBS; CS: healthy mice supplemented with a daily dose of 150 mg/kg bodyweight of CS disaccharides for 16 days; M: mice subjected to exhaustive exercise stress + PBS; S: the stressed mice supplemented with a daily dose of 150 mg/kg bodyweight of CS disaccharides for 16 days. *P < 0.05; **P < 0.01.

The mice experiencing exhaustive exercise stress had a significantly lowered body weight and feed intake as expected (Fig. 1). One day after exhaustive exercise, the mice had a significant reduction in feed intake (*P* < 0.01). After a short period of adaptation, the feed intake started to bounce back in the mice experiencing the stress. A notable decrease in body weight in the mice with the stress was seen after day 3 (*P* < 0.05). This reduction in body weight remained significant during the much of experimental duration until the conclusion of the experiment at day 16. The exercise stressed mice supplemented with CS disaccharides for 16 days did not appear to have a notable effect on both bodyweight and feed intake. Neither did CS disaccharides affect these two physiological parameters in healthy mice (Fig. 1). However, the exercise stress resulted in a 39% reduction in intestinal villus length, from 1154.8 µm in the healthy mice to 707.4 µm in the stressed mice (*P* < 0.01; Fig. 2) while intestinal crypt depth was not significantly impacted (data not shown). As a result, the stress significantly altered the villus to crypt ratio (V/C, Fig. 2). Intriguingly, the stressed mice supplemented with CS disaccharides for 16 days had a significant increase in both villus length (*P* < 0.01) and V/C ratio (*P* < 0.05). CS disaccharides supplementation was able to restore repressed villus length from 707.4 µm in the stressed mice to the baseline level observed in the healthy mice (1062.6 µm, *P* < 0.05), suggesting that CS disaccharides may have potential to increase intestinal absorption.

**Figure 3:**
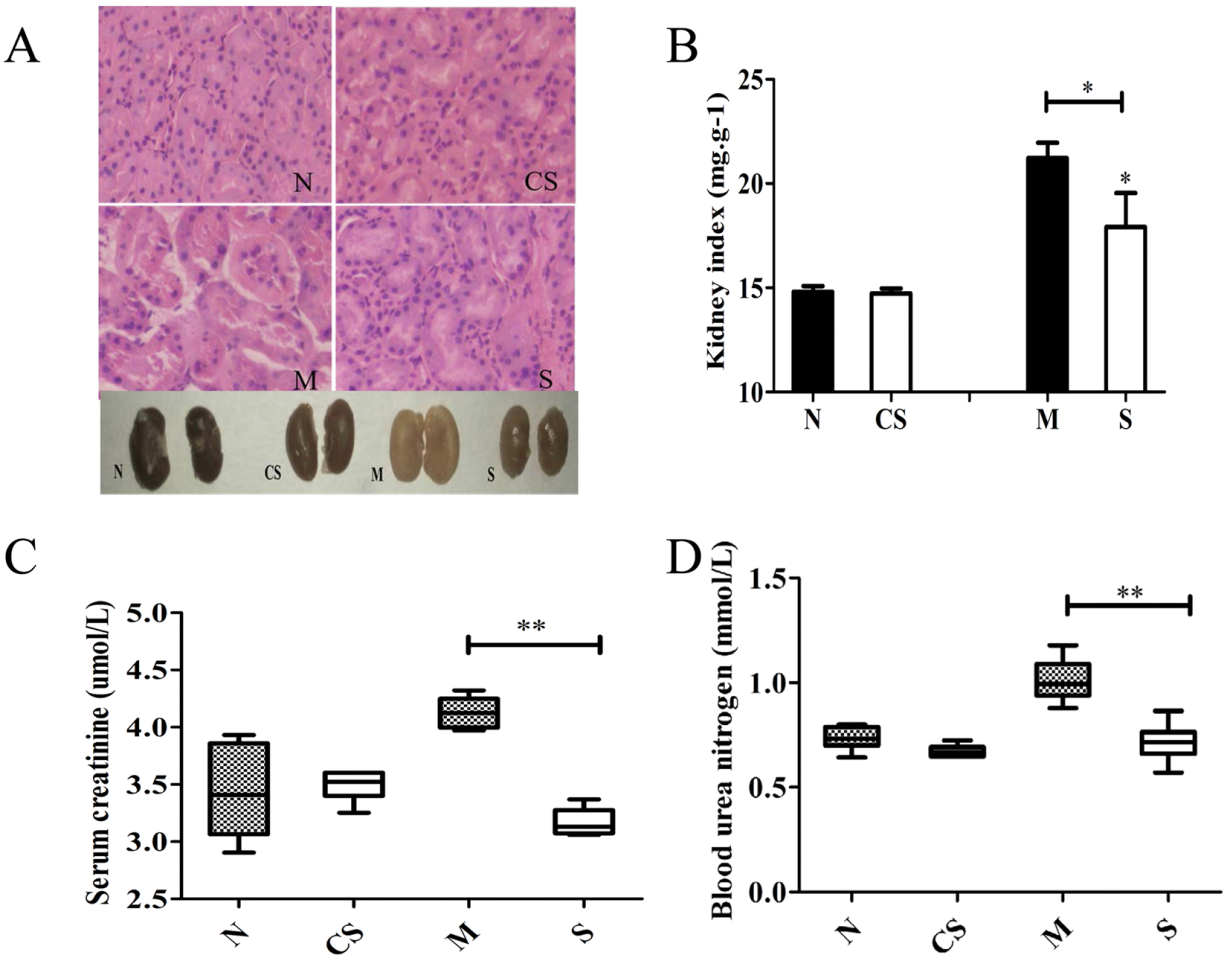
Kidney morphology and blood parameters. (**A**) Kidney morphology (the cortex, 40x); (**B**) Kidney index; (**C**) Serum creatinine levels. (**D**) Blood urea nitrogen levels. N: healthy mice + PBS; CS: healthy mice supplemented with a daily dose of 150 mg/kg bodyweight of CS disaccharides for 16 days; M: mice subjected to exhaustive exercise stress + PBS; S: the stressed mice supplemented with a daily dose of 150 mg/kg bodyweight of CS disaccharides for 16 days. *P < 0.05; **P < 0.01.

As Fig. 3 shows, the stress altered kidney morphology and tissue structure (Fig. 3A), especially the kidney cortex, and significantly increased the kidney weight (mg) to total bodyweight (g) ratio or kidney index (Fig. 3B, *P* < 0.001). The indices of liver and thymus were also significantly impacted by the exercise stress (data not shown). Moreover, CS disaccharides were able to partially restore the kidney index to the normal level (Fig. 3B, *P* < 0.05). Serum creatinine (Fig. 3C) and blood urea nitrogen (Fig. 3D) levels followed the similar trend, significantly increased by the exercise stress and restored to the normal baseline levels by CS disaccharides under the stressed condition. However, CS disaccharides did not appear to have any effect on kidney index as well as the two blood parameters tested in healthy mice. In addition, we did not observe any effect of CS disaccharide supplementation on the indices of liver and thymus under the stressed condition either.

### Potential anti-inflammatory properties of CS disaccharides

**Figure 4:**
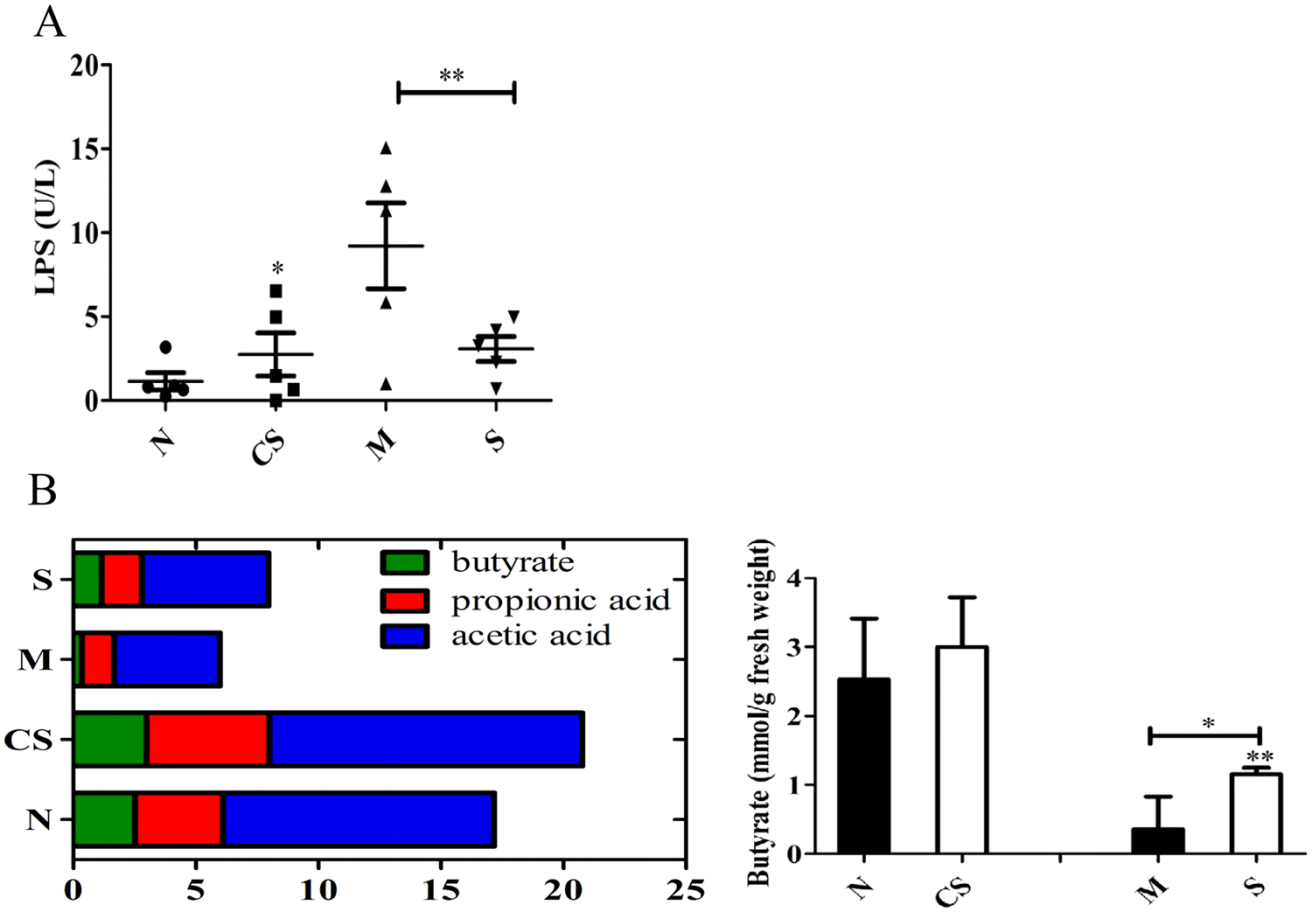
Blood lipopolysaccharides (LPS) and fecal short-chain fatty acid (SCFA) levels. (**A**) Blood LPS; (**B**) total SCFA and butyrate concentrations. N: healthy mice + PBS; CS: healthy mice supplemented with a daily dose of 150 mg/kg bodyweight of CS disaccharides for 16 days; M: mice subjected to exhaustive exercise stress + PBS; S: the stressed mice supplemented with a daily dose of 150 mg/kg bodyweight of CS disaccharides for 16 days. *P < 0.05; **P < 0.01.

The exhaustive exercise stress significantly elevated blood LPS level, from the baseline (1.15 U/L) to 9.21 U/L (Fig. 4A, *P* < 0.05). CS disaccharide supplementation was able to lead to a ~67% reduction in blood LPS levels in the stressed mice (*P* < 0.05).

The exercise stress significantly repressed total short-chain fatty acids (SCFA) in feces (Fig. 4B, *P* < 0.01). While the stress induced a significant decrease in the fecal butyrate level, CS disaccharide supplementation significantly increased the fecal total SCFA (*P* < 0.01). Of note, CS disaccharides significantly increased the fecal butyrate level in the stressed mice (Fig. 4B). Under the healthy condition, CS disaccharides appeared to increase fecal butyrate by approximately 20%, not to a level that is statistically significant.

### Exhaustive exercise stress induced a profound change in the gut microbiome

The mean number of Operational Taxonomic Units (OTU) per sample identified in this study was 729.67 ± 321.34 (mean ± sd; *N* = 30; see Supplementary info). Approximately 760 OTU were detected at least once in each of the healthy control mice, including 60 of the 89 named species identified in the study. 160 OTU were present in each of the 30 samples tested and may represent the core microbiome. The OTU comprising of the core microbiome represented 74.25% of all sequences in the healthy control group, likely contributing to the basic function of the microbiome. Among the 25 most abundant OTU in the healthy group (with the relative abundance >1.0%), 21 belonged to the core microbiome. An OTU belonging to *Bacteroides acidifaciens* (GreenGene ID# 356164) and an OTU assigned to the genus *Lactococcus* (GreenGene ID# 586387) were among the most abundant. As Table 1 shows, neither exhaustive exercise nor the CS disaccharide treatment had any impact on various microbial diversity indices. For example, Chao1 and Shannon indices remained unchanged by the stress.

**Table 1:** Chondroitin sulfate disaccharides as a supplement did not appear to affect gut microbial diversity in mice.

Diversity Indices	N	M	S	CS
Total species	763.00 ± 55.10	734.50 ± 52.92	714.00 ± 60.49	691.50 ± 108.35
Richness	165.47 ± 11.96	159.29 ± 11.48	154.83 ± 13.13	149.93 ± 23.54
Evenness	0.66 ± 0.04	0.65 ± 0.06	0.65 ± 0.07	0.62 ± 0.07
PD Whole Tree	44.04 ± 3.45	42.11 ± 2.32	40.00 ± 2.04	40.73 ± 3.18
Chao1	1024.35 ± 88.16	964.88 ± 86.40	902.62 ± 80.84	918.18 ± 110.41
Shannon	6.29 ± 0.40	6.23 ± 0.57	6.20 ± 0.74	5.84 ± 0.79
Simpson (inverse)	0.97 ± 0.01	0.97 ± 0.02	0.96 ± 0.04	0.95 ± 0.04

N: healthy mice + PBS; M: mice subjected to exhaustive exercise stress + PBS; CS: healthy mice supplemented with a daily dose of 150 mg/kg bodyweight of CS disaccharides for 16 days. S: the stressed mice supplemented with a daily dose of 150 mg/kg bodyweight of CS disaccharides for 16 days. The numbers represented mean ± sd.

Exhaustive exercise altered approximately 10% of the fecal microbial community. The abundance of 76 OTU was significantly impacted by exhaustive exercise using Linear discriminant analysis (LDA) Effect Size algorithm (LEfSe)^20^ at a cutoff of absolute log_10_ LDA scores >2.0. Among them, 47 OTU were repressed in abundance by the exercise while 29 OTU were elevated. Select OTU impacted by exhaustive exercise were listed in Table 2. For example, the abundance of at least 10 OTU assigned to the family S24-7 was significantly repressed by the exercise (Table 2). On the other hand, the abundance of an OTU (GreenGene ID# 275069), belonging to the order Clostridiales, was elevated 4 fold by exhaustive exercise (log_10_ LDA = 3.91).

**Table 2:** OTU displaying significantly different abundance between healthy (N) and stressed (M) conditions.

OTU ID	N	M	LDA score	Annotation
342962	1.9728 ± 0.7742	1.0770 ± 0.8516	3.61	Bacteroidetes; Bacteroidia; Bacteroidales; S24-7
416078	1.3081 ± 1.3847	0.1724 ± 0.2778	3.79	Bacteroidetes; Bacteroidia; Bacteroidales; S24-7
384555	1.2773 ± 0.7131	0.4133 ± 0.3167	3.63	Bacteroidetes; Bacteroidia; Bacteroidales; S24-7
288912	1.2549 ± 1.1602	0.3750 ± 0.5008	3.70	Proteobacteria; γ-proteobacteria; Pseudomonadales; Moraxellaceae; Acinetobacter
460953	1.1114 ± 0.3526	0.3462 ± 0.3305	3.57	Bacteroidetes; Bacteroidia; Bacteroidales; S24-7
215897	0.6294 ± 0.2129	0.1508 ± 0.1360	3.36	Bacteroidetes; Bacteroidia; Bacteroidales; S24-7
379505	0.4816 ± 0.2021	0.0437 ± 0.0452	3.32	Bacteroidetes; Bacteroidia; Bacteroidales; S24-7
275069	0.4724 ± 0.4644	1.9150 ± 1.6701	3.91	Firmicutes; Clostridia; Clostridiales
461487	0.2300 ± 0.4588	0.0171 ± 0.0306	3.12	Firmicutes; Clostridia; Clostridiales
4482227	0.2105 ± 0.1505	0.0719 ± 0.0507	2.81	Firmicutes; Bacilli; Lactobacillales; Leuconostocaceae; Leuconostoc; mesenteroides
321972	0.1176 ± 0.0714	0.0328 ± 0.0324	2.66	Bacteroidetes; Bacteroidia; Bacteroidales; Bacteroidaceae; Bacteroides; acidifaciens
341448	0.1120 ± 0.0451	0.0117 ± 0.0156	2.69	Bacteroidetes; Bacteroidia; Bacteroidales; S24-7
338887	0.1047 ± 0.0755	0.6937 ± 0.4773	3.49	Firmicutes; Clostridia; Clostridiales; Ruminococcaceae; Oscillospira
619817	0.1011 ± 0.0716	0.0416 ± 0.0199	2.47	Firmicutes; Clostridia; Clostridiales; Lachnospiraceae; Anaerostipes
961009	0.0937 ± 0.0974	0.0224 ± 0.0193	2.57	Proteobacteria; γ-proteobacteria; Pseudomonadales; Moraxellaceae; Acinetobacter; johnsonii
141564	0.0874 ± 0.0805	0.0145 ± 0.0125	2.57	Proteobacteria; γ-proteobacteria; Pseudomonadales; Pseudomonadaceae
272834	0.0760 ± 0.1566	0.1183 ± 0.0972	2.51	Firmicutes; Clostridia; Clostridiales; Ruminococcaceae; Oscillospira
549991	0.0597 ± 0.0614	0.0127 ± 0.0085	2.32	Firmicutes; Bacilli; Lactobacillales; Lactobacillaceae; Lactobacillus
252339	0.0590 ± 0.0324	0.0201 ± 0.0106	2.27	Firmicutes; Bacilli; Lactobacillales; Leuconostocaceae; Leuconostoc; mesenteroides
264534	0.0495 ± 0.1159	0.0005 ± 0.0012	2.39	Bacteroidetes; Bacteroidia; Bacteroidales; S24-7
304408	0.0486 ± 0.0225	0.0051 ± 0.0064	2.34	Bacteroidetes; Bacteroidia; Bacteroidales; S24-7
397065	0.0272 ± 0.0453	0.2363 ± 0.2557	2.99	Firmicutes; Clostridia; Clostridiales; Lachnospiraceae; Coprococcus
315669	0.0268 ± 0.0099	0.0023 ± 0.0027	2.16	Bacteroidetes; Bacteroidia; Bacteroidales; S24-7
248902	0.0217 ± 0.0434	0.0008 ± 0.0013	2.08	Firmicutes; Bacilli; Turicibacterales; Turicibacteraceae; Turicibacter
466811	0.0130 ± 0.0139	0.1053 ± 0.1420	2.65	Firmicutes; Clostridia; Clostridiales
311961	0.0128 ± 0.0186	0.0360 ± 0.0267	2.11	Firmicutes; Clostridia; Clostridiales; Ruminococcaceae; Anaerotruncus
276050	0.0064 ± 0.0067	0.0808 ± 0.0757	2.58	Firmicutes; Clostridia; Clostridiales
817176	0.0053 ± 0.0032	0.0026 ± 0.0014	2.05	Firmicutes; Bacilli; Lactobacillales; Streptococcaceae; Streptococcus
263272	0.0014 ± 0.0016	0.0000 ± 0.0000	2.29	Firmicutes; Clostridia; Clostridiales; Ruminococcaceae; Ruminococcus
266210	0.0008 ± 0.0010	0.0000 ± 0.0000	2.17	Firmicutes; Clostridia; Clostridiales; Veillonellaceae; Megasphaera

The numbers represented relative abundance (mean ± sd). The significance threshold is the absolute log_10_ Linear Discriminant Analysis (LDA) score > 2.0.

**Figure 5:**
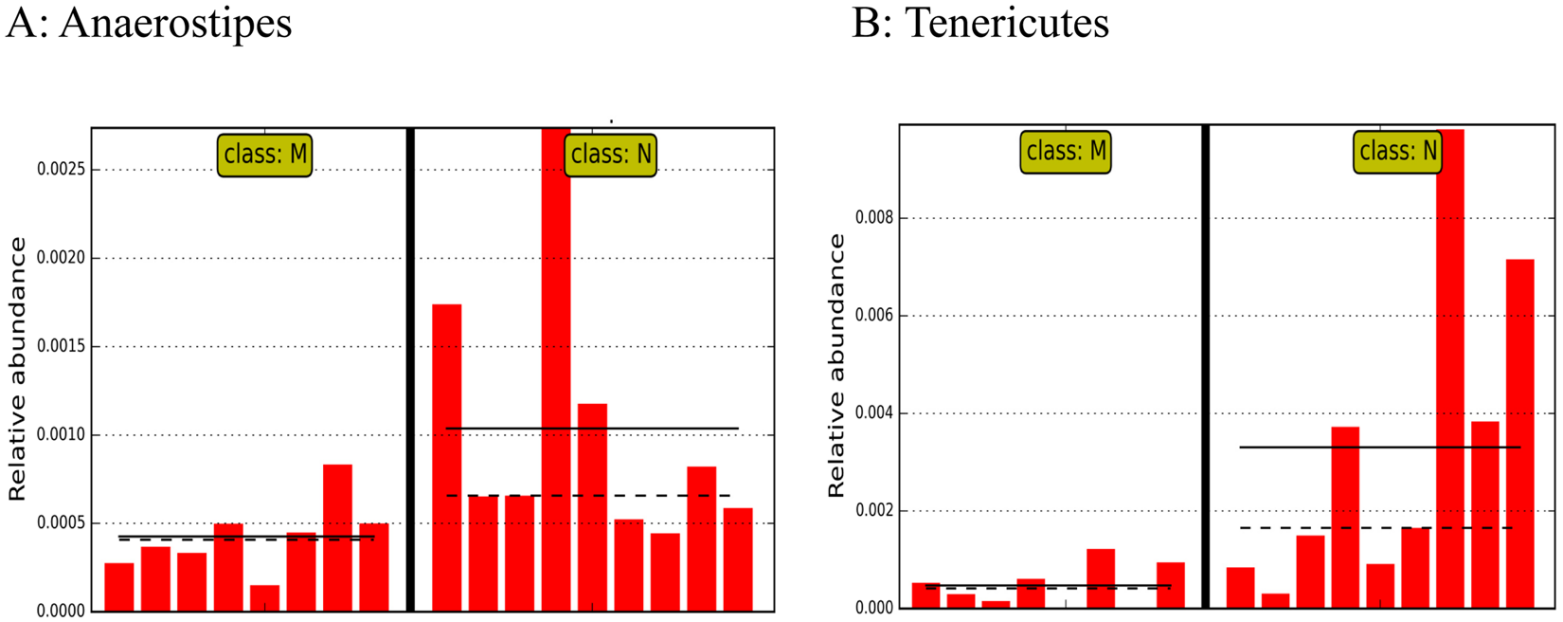
Microbial taxa displaying significant differences in relative abundance between healthy and exhaustive exercise induced stressed mice. (**A**) Anaerostipes. (**B**) Tenericutes. N: healthy mice + PBS; M: mice subjected to exhaustive exercise stress + PBS. Straight line: group mean abundance. Dotted Line: median.

At the genus level, at least 4 genera was significantly impacted by exhaustive exercise. The abundance of *Aeromicrobium*, *Anaerostipes* (Fig. 5), and *Turicibacter* was significantly decreased by the exercise while the abundance of the genus *Anaerotruncus* as well as the family that this genus belongs to, Ruminococcaceae, was elevated by exhaustive exercise. At the order level, Actinobacteria was significantly decreased by the exercise. Similarly, the phylum Tenericutes underwent a significant reduction in relative abundance during the exercise (Fig. 5).

### CS disaccharides modulated gut microbiome under both healthy and stressed conditions

CS disaccharides as a dietary supplement affected the abundance of 99 and 53 OTU under healthy and exhaustive exercise stressed conditions, respectively (Supplementary data). Specifically, CS increased the abundance of 44 and 32 OTU under healthy and stressed conditions, respectively. Intriguingly, at least 7 OTU displayed a unidirectional change in their abundance under both conditions. For example, OTU#321972, assigned to *Bacteroides acidifaciens*, was significantly increased by CS disaccharides under both healthy and stressed conditions. In healthy mice, CS disaccharides induced a 2-fold increase in its abundance, from 0.12% to 0.22%. However, the changes in the abundance of *B*, *acidifaciens* induced by CS disaccharides became more profound under the stressed condition, up by ~8-fold. The exercise itself had a suppressive effect on the abundance of this OTU (Table 3). Likewise, an OTU belonging to the family S24-7 (GreenGene ID# 266976) was significantly increased in its abundance by CS disaccharides under both conditions. An OTU (GreenGene id# 4390359) assigned to *Lysinibacillus boronitolerans*, while rare, was also significantly increased by CS disaccharides under both conditions.

**Table 3:** Operational Taxonomic Units (OTU) significantly impacted by CS under both healthy and stressed conditions in mice.

OTU_ID (GreenGene)	Annotation	N	CS	M	S	Significant
321972	*Bacteroides acidifaciens*	0.1176 ± 0.0714	0.2173 ± 0.0834	0.0328 ± 0.0324	0.2421 ± 0.2454	abc
1135084	*Bacteroides sp*,	0.0007 ± 0.0014	0.2006 ± 0.2291	0.0000 ± 0.0000	0.0012 ± 0.0010	ab
4020502	*Bacteroides sp*,	0.0004 ± 0.0009	0.0609 ± 0.0778	0.0000 ± 0.0000	0.0017 ± 0.0016	ab
4390359	*Lysinibacillus boronitolerans*	0.0000 ± 0.0000	0.0031 ± 0.0047	0.0000 ± 0.0000	0.0016 ± 0.0012	ab
930014	*Pseudochrobactrum sp*,	0.0014 ± 0.0023	0.0041 ± 0.0011	0.0000 ± 0.0000	0.0007 ± 0.0010	ab
827522	[*Clostridiales*]	0.0125 ± 0.0324	0.1545 ± 0.1663	0.0047 ± 0.0100	0.1672 ± 0.1160	ab
266976	[*S24*-*7*]	0.0247 ± 0.0335	0.1305 ± 0.1459	0.0299 ± 0.0247	0.0729 ± 0.0516	ab

The numbers denote the relative abundance (mean ± SD). N: healthy mice + PBS; M: mice subjected to exhaustive exercise stress + PBS; CS: healthy mice supplemented with a daily dose of 150 mg/kg bodyweight of CS disaccharides for 16 days. S: the stressed mice supplemented with a daily dose of 150 mg/kg bodyweight of CS disaccharides for 16 days. Significant means that the abundance of the OTU that was changed at a cutoff value of the absolute log10 LDA scores > 2.0 between the two contrast groups using Linear Discriminant Analysis (LDA) Effect Size (LEfSe) algorithm (a = CS vs N; b = S vs M; c = M vs N).

**Figure 6:**
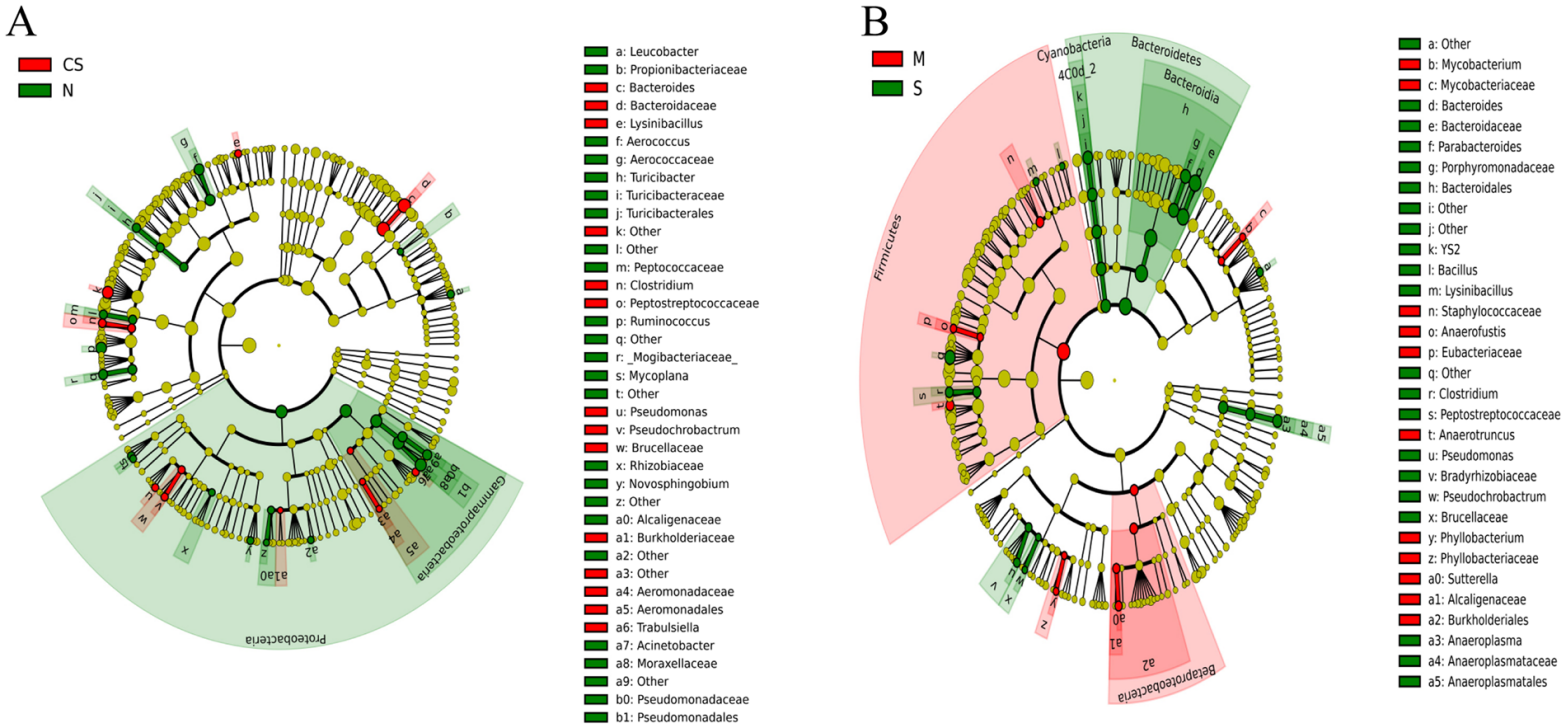
Graphical representation of the taxa with significantly different abundance induced by CS disaccharides under healthy (**A**) and exhaustive exercise-induced stress conditions (**B**) in a phylogenetic tree format. The statistical significance cutoff values are an absolute Linear Discriminant Analysis LDA score log 10 ≥ 2.0.

**Figure 7:**
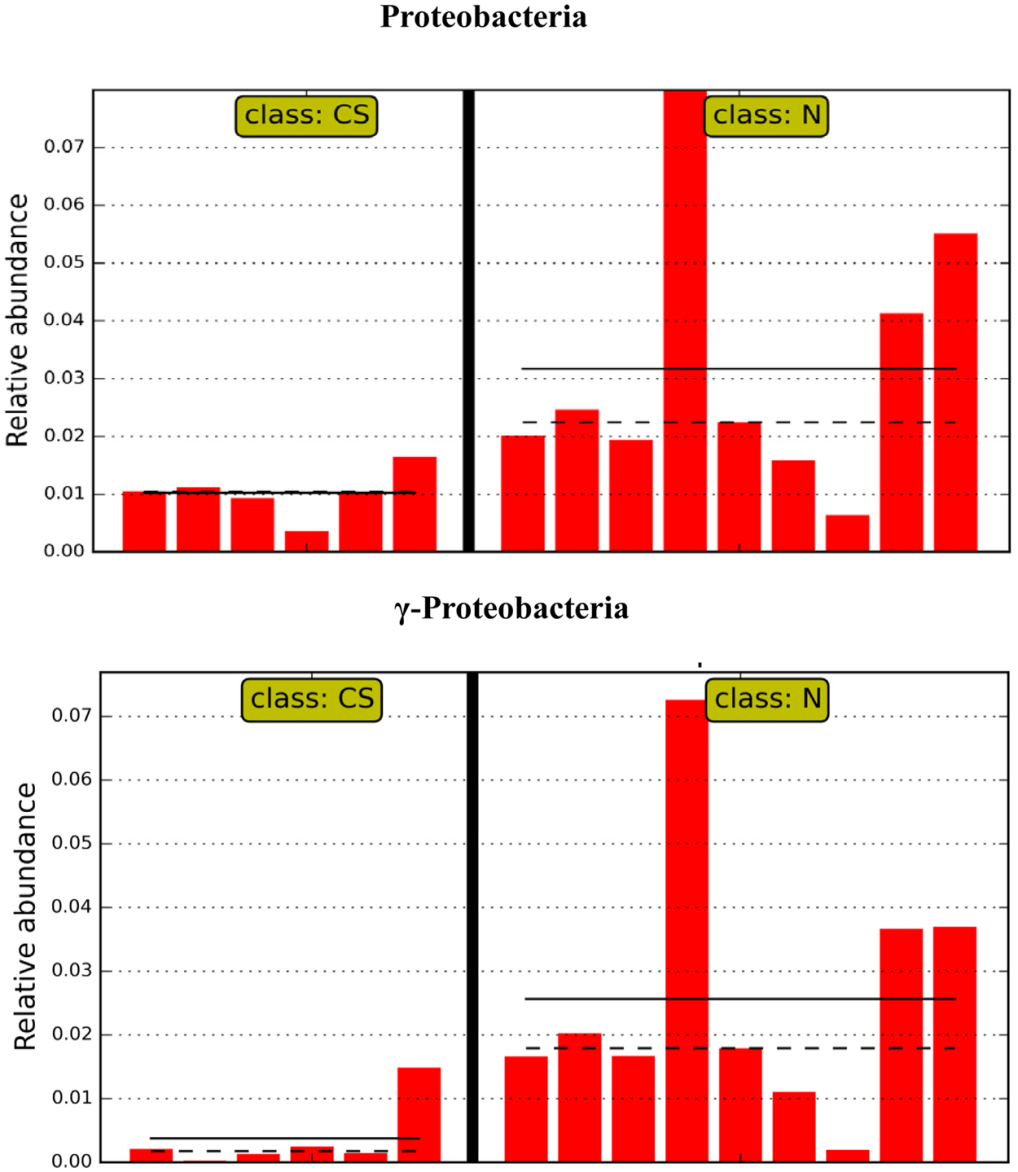
Microbial taxa significantly impacted by CS disaccharides in healthy mice. (**A**) Proteobacteria. (**B**) γ-Proetobacteria. N: healthy mice + PBS; CS: healthy mice supplemented with a daily dose of 150 mg/kg bodyweight of CS disaccharides for 16 days. Straight line: group mean abundance. Dotted Line: median.

**Figure 8:**
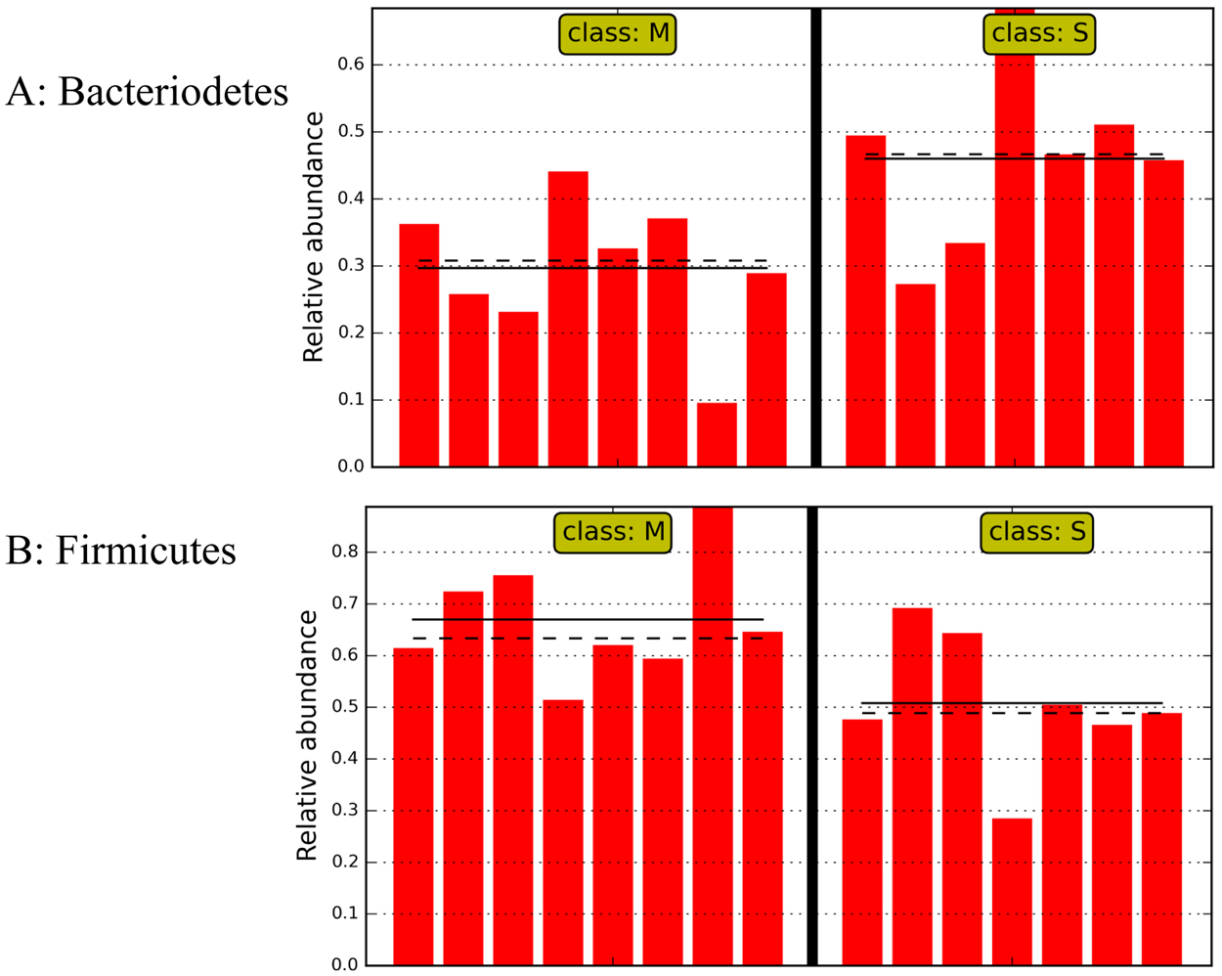
The two most abundant phyla, Bacteroidetes and Firmicutes, were significantly impacted by CS disaccharide supplement in the mice subjected to exhaustive exercise stress. M: mice subjected to exhaustive exercise stress + PBS; S: the stressed mice supplemented with a daily dose of 150 mg/kg bodyweight of CS disaccharides for 16 days. Straight line: group mean abundance. Dotted Line: median.

The changes induced by CS disaccharides at higher taxon levels appeared to be profound as well. 40 and 38 taxa were significantly impacted by CS disaccharides under healthy and stressed conditions, respectively (Fig. 6). Under the healthy condition, at least 13 named genera were significantly affected. CS disaccharides tended to increase the abundance of *Bacteroides*, *Clostridium*, *Lysinibacillus*, *Pseudochrobactrum*, *Pseudomonas*, and *Trabulsiella*. On the other hand, the phylum Proteobacteria (Fig. 7), especially one of its class-level constituents, γ-Proteobacteria (Fig. 7), was significantly decreased by CS, from 3.1% in the healthy controls to 1.0%. Under the stressed condition, two most abundant phyla, Bacteroidetes and Firmicutes, were significantly impacted by CS disaccharides (Fig. 8). The abundance of Firmicutes was reduced from 67.5% in the stressed group to 50.8% by CS disaccharides. The similar trend of reduction was also observed under the healthy condition, but not reaching a statistically significant level (from 62.3% in the healthy control to 57.3% in CS group). The reduction in the Firmicutes abundance followed a concomitant increase in the second most abundant phylum, Bacteroidetes. CS disaccharides induced a significant increase in the abundance of Bacteroidetes, from 29.2% to 46.0% under the stressed condition (log_10_ LDA > 2.0), compared to an insignificant increase from 32.7% to 40.5% under the healthy condition. At the class level, γ-Proteobacteria remained unchanged by CS disaccharides under the stressed condition, unlike in healthy mice. However, CS disaccharides significantly reduced the abundance of β-Proteobacteria under stress.

At least 10 genera, *Anaerofustis*, *Anaerostipes*, *Anaerotruncus*, *Butyricimonas*, *Cloacibacterium*, *Clostridium*, *Coprococcus*, *Faecalibacterium*, *Megasphaera*, and *Roseburia*, known to harbor butyrate-producing bacteria, were detected in this study. Collectively, this functional group represented approximately 1.6% of all sequences in the fecal microbiome of the healthy control mice. CS disaccharide treatments appeared to result in a slightly higher level (not statistically significant) of butyrate-producing bacteria in the community, from 1.6% to 2.1% and 2.1% to 2.2% under healthy and stressed conditions, respectively.

### Microbial pathways impacted by CS disaccharides

Using the Phylogenetic Investigation of Communities by Reconstruction of Unobserved States algorithm (PICRUSt)^21^, we were able to infer biological function in the fecal microbial community impacted by CS disaccharides. Under the stressed condition, CS disaccharides significantly increased the abundance of 49 KEGG (Kyoto Encyclopedia of Genes and Genomes) enzymes while 15 KEGG enzyme classes were repressed by the treatment. For example, CS disaccharide supplementation led to a 2-fold increase in the abundance of polysaccharide export outer membrane protein (K01991, log_10_ LDA score = 2.2086). The abundance of glucosamine-6-phosphate deaminase [EC:3.5.99.6, K02564], dipeptidyl-peptidase 4 [EC:3.4.14.5, K01278], and outer membrane factor, OMF family (K03287) was also significantly increased by the supplementation. On the other hand, the abundance of multiple sugar transport system permease protein (K10118) and antibiotic transport system ATP-binding protein (K09687) was repressed by the CS disaccharide treatment under the stressed condition. Intriguingly, the abundance of N-acetylmuramoyl-L-alanine amidase [EC:3.5.1.28, K01447] was significantly increased by CS disaccharides under both healthy and stressed conditions. Collectively, 364 OTU possess the genes encoding K01447 in the healthy mice supplemented with CS (mean ± sd = 195.5 ± 22.1 OTU per sample). Among them, two OTU contributed significantly to the abundance of K01447 in both healthy and stressed mice supplemented with CS. One OTU (GreenGene ID#356164) is assigned to *B*, *acidifaciens* while another (ID# 228601) also belongs to the genus *Bacteroides*. Both OTU were the components of the core microbiome and significantly increased by CS under both conditions. These two OTU contributed to 62% and 19% of the K01447 abundance in healthy and stressed mice supplemented with CS, respectively. Other OTU contributing significantly to K01447 abundance included those assigned to S24-7, such as GreenGene ID# 217100 and # 275339, and from the genus *Streptococcus* (ID# 579608).

CS disaccharide supplementation had a broad impact on the basic biological functions at the pathway level in the fecal microbial community. As Table 4 shows, CS disaccharides significantly affected some of very basic biological function categories. For example, energy metabolism, ABC transporters, two-component system, and transcription machinery were among the categories significantly impacted. Of note, CS disaccharides significantly increased the hits assigned to GAG degradation and LPS biosynthesis while repressing ABC transporter under the stressed condition. Moreover, the capacity for secretion system was significantly decreased under both healthy and stressed conditions.

**Table 4:** Pathways significantly impacted by CS in mice.

Pathway	CS	N	S	M	LDA score	Significant
ABC transporters	3.04 ± 0.38	3.28 ± 0.23	2.96 ± 0.49	3.44 ± 0.35	3.3934	b
Alanine, aspartate and glutamate metabolism	1.09 ± 0.05	1.05 ± 0.03	1.11 ± 0.08	1.03 ± 0.04	2.6171	b
Amino acid related enzymes	1.47 ± 0.04	1.46 ± 0.03	1.46 ± 0.03	1.43 ± 0.04	2.2254	b
Amino sugar and nucleotide sugar metabolism	1.53 ± 0.03	1.47 ± 0.06	1.55 ± 0.11	1.49 ± 0.05	2.4525	a
Arginine and proline metabolism	1.23 ± 0.06	1.25 ± 0.04	1.27 ± 0.01	1.24 ± 0.03	2.2171	b
Cell cycle - Caulobacter	0.52 ± 0.02	0.50 ± 0.02	0.50 ± 0.01	0.48 ± 0.02	2.0435	b
DNA repair and recombination proteins	2.86 ± 0.10	2.79 ± 0.10	2.79 ± 0.06	2.72 ± 0.08	2.5630	b
Energy metabolism	0.94 ± 0.13	0.93 ± 0.08	0.97 ± 0.04	0.89 ± 0.08	2.6055	b
Fructose and mannose metabolism	0.94 ± 0.02	0.89 ± 0.03	0.93 ± 0.04	0.90 ± 0.02	2.3932	a
Glycerophospholipid metabolism	0.54 ± 0.01	0.51 ± 0.02	0.51 ± 0.01	0.51 ± 0.02	2.1176	a
Glycosaminoglycan degradation	0.13 ± 0.04	0.11 ± 0.02	0.16 ± 0.06	0.11 ± 0.03	2.4161	b
Glycosphingolipid biosynthesis - ganglio series	0.09 ± 0.03	0.08 ± 0.02	0.11 ± 0.04	0.07 ± 0.02	2.3429	b
Glycosphingolipid biosynthesis - globo series	0.18 ± 0.04	0.18 ± 0.03	0.21 ± 0.04	0.18 ± 0.02	2.2081	b
Lipid biosynthesis proteins	0.58 ± 0.02	0.57 ± 0.02	0.58 ± 0.02	0.55 ± 0.03	2.1402	b
Lipopolysaccharide biosynthesis	0.24 ± 0.07	0.22 ± 0.04	0.28 ± 0.07	0.20 ± 0.05	2.6038	b
Secretion system	1.06 ± 0.06	1.14 ± 0.05	1.06 ± 0.07	1.16 ± 0.03	2.7147	ab
Transcription machinery	0.96 ± 0.11	0.95 ± 0.05	1.04 ± 0.05	0.96 ± 0.04	2.5882	b
Translation factors	0.56 ± 0.03	0.54 ± 0.03	0.54 ± 0.02	0.52 ± 0.03	2.1810	b
Two-component system	1.29 ± 0.17	1.45 ± 0.15	1.40 ± 0.17	1.56 ± 0.16	2.9381	b

The numbers denote the relative abundance (mean ± SD). N: healthy mice + PBS; M: mice subjected to exhaustive exercise stress + PBS; CS: healthy mice supplemented with a daily dose of 150 mg/kg bodyweight of CS disaccharides for 16 days. S: the stressed mice supplemented with a daily dose of 150 mg/kg bodyweight of CS disaccharides for 16 days. Significant means that the abundance of the hits assigned to a given pathway that was changed at a cutoff value of the absolute log10 LDA scores > 2.0 between the two contrast groups using Linear Discriminant Analysis (LDA) Effect Size (LEfSe) algorithm (a = CS vs N; b = S vs M; c = M vs N).

## Discussion

Exercise is known to have an important impact on the microbial diversity and composition of the gut microbiome^22, 23^. While exercise conferring numerous health benefits, the interplay among exercise, energy metabolism, and host immunity is extremely complicated and poorly understood. Intense and prolonged exercise tends to have a negative effect on gut integrity. Gastrointestinal symptoms, such as nausea, heartburn, diarrhea, and bleeding, are common during exhaustive exercise, due to various mechanic, ischemic, and nutritional factors. Recent data show that diet and exercise act orthogonally on the gut microbiome and exercise does not rescue the negative effect of high-fat-diet induced alterations on the gut microbial composition^24^. Our data show that at the phylum level, exhaustive exercise significantly reduced the abundance of Tenericutes, which confirmed a previous observation that both diet and exercise independently decrease its relative abundance^24^. At the class level, exhaustive exercise significantly reduced the abundance of Actinobacteria. A similar reduction in the relative abundance of the phylum Actinobacteria was also observed by exercise in the mice fed a low-fat diet^25^. Our findings demonstrated that exhaustive exercise altered the abundance of approximately 76 OTU in the fecal microbiome, representing approximately 10% of OTU in a given fecal microbial community, in agreement with a previous study that the massive shifts in the gut microbiome induced by exercise are at nearly the same magnitude as diet. As Table 2 shows, the abundance of at least 10 OTU belonging to the family S24-7 was significantly reduced by exhaustive exercise. Common GI disorders alone can have a significant impact on fecal microbial community. For example, significant differences in the fecal microbiome composition between healthy and diarrheic cats can be readily detected^26^. The relative abundance of the phylum Proteobacteria and the class Bacilli as well as the genera *Clostridium*, *Escherichia*, and *Streptococcus* is significantly increased in diarrheic cats. Comparing to their respective healthy controls, exhaustive exercise stress-induced changes in the fecal microbial community bear little resemblance to the changes induced by diarrhea, suggesting that the observed changes in the gut microbiome composition in the exhaustive exercise-induced stress animals are unlikely due to the stress-associated GI disturbances, such as diarrhea.

The increased abundance of the phylum Proteobacteria has been suggested as a marker for an unstable microbial community and a risk factor of human disease^27^. An elevated Proteobacteria level is frequently observed in patients with metabolic disorders^27, 28^. Furthermore, a transient high level of Proteobacteria is a striking feature of the gut microbiota of Toll-like receptor 5 deficient mice that display low-grade inflammation or a colitic phenotype^29^. Recent studies support a causative role of Proteobacteria in intestinal inflammation^30, 31^. In this study, we observed a significant reduction in the abundance of the phylum Proteobacteria induced by CS disaccharides, from 3.10% (±2.20%, sd) in healthy mice to 1.03% (±0.41%, sd) in the healthy mice supplemented with CS disaccharides (absolute log_10_ LDA value = 4.096). A similar CS disaccharide-induced reduction was also observed in the stressed mice; and the abundance of Proteobacteria was decreased from 1.98% in the stressed mice to 1.44% in the stressed mice with CS supplementation. At the class level, the abundance of γ-Proteobacteria and β-Proteobacteria was significantly reduced by CS disaccharides in healthy and stressed mice, respectively (log_10_ LDA value = 4.1023 and 2.6161, respectively). For example, the abundance of the class γ-Proteobacteria was decreased from 2.50% in healthy mice to 0.38% in the healthy mice supplemented with CS disaccharides. Our findings suggest that CS disaccharides may possess anti-inflammatory properties by repressing the prevalence of inflammatory Proteobacteria.

Our data show that fecal total SCFA level was decreased by approximately 60%, from 18.94 mmol/g fresh feces in healthy mice to 7.61 mmol/g in the mice that experienced exhaustive exercise stress (Fig. 4B, *P* < 0.05). Among four SCFA measured, the butyrate level was repressed from 2.53 mmol/g in healthy mice to a level barely detectable, 0.35 mmol/g, in the stressed mice. CS disaccharides increased total SCFA as well as butyrate levels under both healthy and stressed conditions. For example, butyrate levels were increased over 2-fold by CS disaccharides, from 0.35mmo/g in the stressed mice to 1.16 mmol/g in the stressed mice supplemented with CS disaccharides (*P* < 0.01). While the supplement also tended to increase butyrate in the feces of healthy mice, the effect was less apparent.

At least 10 genera known to harbor butyrate-producing bacteria, *Anaerofustis*, *Anaerostipes*, *Anaerotruncus*, *Butyricimonas*, *Cloacibacterium*, *Clostridium*, *Coprococcus*, *Faecalibacterium*, *Megasphaera*, and *Roseburia*, were detected in murine fecal microbiome in our dataset. The butyrate-producing bacteria as a functional group were less predominant and represented approximately 1.56% of the microbial community. CS disaccharides led to a nominal increase in butyrate-producing bacterial populations under both healthy and stressed conditions. The increase was not statistically significant, nevertheless. Among the factors contributing to butyrate production, the abundance of butyrate-producing bacteria, availability of substrates, and intestinal transit time play important roles. Butyrate is a potent anti-inflammatory molecule^32, 33^. Our findings demonstrated that CS disaccharides were likely butyrogenic substrates. A significant increase in the fecal butyrate level by CS disaccharides may represent one of the contributing factors to their potential anti-inflammatory properties. In an *in vitro* model, chondroitin 4-sulfate (C4S) is able to reduce inflammation mediators, such as TNFα, IL-1β, IL6, MMP1, and nitric oxide (NO), and apoptosis in LPS-treated mouse chondrocytes in a dose-dependent manner^34^. C6S exerts a similar anti-inflammatory effect but it fails to decrease NO production. Similarly, purified high-sulfur-containing fraction of fish GAG extraction (including CS) results in a significant reduction of IL-1β production in intestinal Caco-2 cells^35^. Direct anti-inflammatory properties of CS may be due to its ability to inhibit NF-κB DNA binding to the nucleus. In addition, CS, due to its polyanionic structure, has been suggested to affect the attachment of both probiotic and pathogenic bacteria to intestinal epithelial cells *in vitro*. Together, our data suggest CS can exert its anti-inflammatory effect in both indirectly and direct manners.

*Bacteroides acidifaciens* is a recently described species predominantly colonized in the murine gut^36^. This obligately anaerobic, Gram-negative species is responsible for promoting IgA production in the large intestine by increasing the number of IgA^+^ B cells^37^. Moreover, *B*, *acidifaciens* possesses stronger immunomodulating activities in the large intestine than other common commensal bacteria, such as *Lactobacillus johnsonii*. A recent study shows that this species is an important host protein forager^38^. *B*, *acidifaciens* population is significantly increased in the feces of Atg7-compromised mice, which display reduced body weight and fat mass than Atg7^f/f^ mice^39^. Furthermore, wild-type C57BL/6 (B6) mice fed with *B*, *acidifaciens* are more likely to gain less weight and fat mass than mice fed with PBS, suggesting this species may have potential for the treatment of diabetes and obesity^39^. Both high-fiber diet and acetate supplementation increase the prevalence of *B*, *acidifaciens* in hypertensive mice^40^. The relative abundance of *B*, *acidifaciens* is also positively correlated with hepatic miR-21 expression and negatively correlated with liver triacylglycerol levels during metabolic adaptation to high-fat diet in mice^41^. There are 5 OTU assigned to *B*, *acidifaciens* in our dataset. Together, this species represented approximately 5.36% of all sequences in healthy control mice and was the most predominant species. The abundance of at least 3 OTU consisting of this species was significantly increased by the supplement in the healthy mice. In the stressed mice, the abundance of this species was increased from 5.87% to 11.44% by CS disaccharides. One of these OTU (GreenGene ID# 321972) was significantly increased under both healthy and stressed conditions by CS disaccharides. In addition, other IgA-stimulating genera, such as *Clostridium*, are also present in the murine gut. Notably, the abundance of *Clostridium* was significantly increased by CS disaccharides under both healthy and stressed conditions. Together, our data suggest that CS disaccharides may act as a bioactive compound to modulate host immunity by enhancing the abundance of certain bacterial species that promote IgA production.

The PICRUSt algorithm allows us to infer functional categories impacted by CS disaccharides in the fecal microbiome^21^. Depending on availability of reference genomes for microorganisms, this tool can achieve relatively high correlations (~0.8) between inferred and metagenomically measured gene contents^21^. While experimental validation is still needed, this approach provides a rapid snapshot on how dietary supplements shape up biological function in the gut microbial community. In this study, we were able to predict dozens of KEGG enzymes that were significantly impacted by CS disaccharides. For example, the abundance of α-glucosidase [EC:3.2.1.20, K01187] and β-galactosidase [EC:3.2.1.23, K01190] was significantly increased by CS disaccharides under healthy and stressed conditions, respectively. Another enzyme, N-acetylmuramoyl-L-alanine amidase [EC:3.5.1.28, K01447], was increased by CS disaccharides under both conditions.

Under healthy condition, the hits assigned to amino sugar and nucleotide sugar metabolism, fructose and mannose metabolism, and glycerophospholipid metabolism were significantly higher in the mice supplemented with CS disaccharides than the mice received PBS only. The increase in these pathways is likely due to increased substrate availability in the gut microbial ecosystem. It is well known that glycan gradients in the gastrointestinal tract are major determinants of the gut microbial composition^42^. CS disaccharides had a significant impact on a broader range of metabolic pathways under the stressed condition than under healthy condition. For example, at least two pathways related to environmental information processing, ABC transporters and two-component systems (TCS), were significantly repressed by CS disaccharides under the stressed condition. Certain ABC transporters and neighboring TCS have coevolved to form self-sufficient detoxification modules against antimicrobial peptides^43^. ABC transporters are frequently co-located and co-regulated with the genes encoding glycoside hydrolases, and, together, they form an important strategy for carbohydrate utilization^42^. Many ABC transporters, such as BceAB-like, are present almost exclusively in the phylum Firmicutes^43^. It is likely that the CS disaccharide-induced decrease in ABC transporters and TCS in the stressed mice resulted from the reduction in the prevalence of the phylum Firmicutes. Indeed, The abundance of Firmicutes underwent a significant reduction, from 67.51% in the feces of the stressed mice to 50.83% in the stressed mice fed with CS disaccharides. A smaller reduction in the Firmicutes abundance was also observed in healthy mice by the supplement, from 62.26% to 57.28%. Among the 43 pathways significantly increased by CS disaccharides in the stressed mice, 23 belonged to the metabolism category, followed by genetic information processing (10 pathways). Amino acid metabolism and carbohydrate and glycan metabolism were predominantly impacted by CS disaccharides in the stressed mice. In contrast to the healthy condition, CS disaccharides appeared to primarily affect environmental information processing in the stressed mice. Taken together, our data suggest that CS disaccharides possess potential to restore altered metabolic function by exhaustive exercise. Our observations underscore the importance of CS disaccharides as a potential bioactive compound to modulate host immunity and restore altered structure and function of the gut microbiome during exhaustive exercise.

## Methods

### Animals and treatment

Thirty male Balb/c mice (18–20 g, 4 weeks old) were obtained from Vital River Laboratory Animal Technology Co., Ltd. (Beijing, China). During the experimental period, mice were housed in a room maintained under a 12 hours light/dark cycle at 24 °C. Mice had free access to fresh water. Mice were fed *ad libitum* a Maintenance Purified Diet (AIN-93M) throughout the experiment period. After a 7-day acclimation period, mice were randomly assigned to 4 groups: Healthy controls + PBS (N), Exhaustive exercise + PBS (M), Healthy mice supplemented with CS disaccharides (CS); and Exhaustive exercise mice supplemented with CS disaccharides (S). In the following 16 days, the N and M groups were given an oral administration of normal saline (PBS) once a day. The CS and S treatment groups of mice were given CS disaccharides with a daily dosage of 150 mg/kg bodyweight by oral gavage. At the end of the feeding period and following an overnight fasting, mice were anaesthetized and killed by cervical dislocation. Blood and feces were collected for subsequent analysis. This study was carried out in strict accordance with the recommendations in the Guide for the Care and Use of Laboratory Animals of the National Institutes of Health. The protocol was approved by the Committee on the Ethics of Animal Experiments of Ocean University of China (SCXK (Jing) 2007-0001).

### Exhaustive exercise protocol

A forced exercise wheel-track treadmill (YLS-10B, Shandong Academy of Medical Sciences, Jinan, China) was used in this study. Animals in both Exhaustive exercise (M) and S groups were subjected to an intensive exercise protocol while the animals in the C and CS groups (healthy groups) were raised without the treadmill exercise. After an initial resting period to collect baseline data, exercise commenced at 20 rpm running speed for 2 days. A recovery and resting period of 5 days was allowed after the 2-day exercise. The exercise-resting cycle was repeated. The mice experienced a total of 6 days’ intensive exercise during the experimental period. The onset of exhaustion was noted. During the exercise, the rest times and running distance were automatically recorded.

### Preparation of CS disaccharides

CS disaccharides were enzymatically prepared in the lab. Briefly, CS was extracted from chicken cartilage using a CS-digesting enzyme purified from *Sphingomonas paucimobilis*, a non-fermenting Gram-negative bacillus cultured in the lab. The crude CS oligosaccharide preparations were further purified by gel filtration chromatography at a flow rate of 1.0 mL/min. The final CS disaccharide preparation used in the study reached a purity of >90% and contained CS-4S (CS-A) and CS-6S (CS-C) at a ratio of 81:16.

### The detection of serum parameters

Serum endotoxin (LPS) levels were measured using ELISA (R&D Systems, Minneapolis, MN, USA). Blood creatinine kinase (CK) activity and urea nitrogen contents were measured using assay kits purchased from Nanjing Jiancheng Bioengineering Institute (Nanjing, China).

### Tissue histology

The intestinal tissue samples were obtained from the ileum. The kidney and the ileum tissue samples were routinely processed using haematoxylin and eosin (HE) stain. Briefly, the samples were fixed in 10% formalin for 24 h, washed with dd water, dehydrated with gradient concentrations of alcohol, and embedded in paraffin. After standard fixation and dehydration processes, the tissue samples were sectioned at a 5-µm thickness and stained with haematoxylin and eosin. The micrographs were taken from the kidney cortex of male mice. The villus length and the crypt depth of the small intestine were measured using a digital image analysis system (Olympus Optical Co. Ltd, Tokyo, Japan).

### Analysis of fecal SCFA

Fecal SCFA contents were determined by gas chromatography according to a published method^44^. Briefly, fecal samples were stored in the −80 °C freezer until use. 1,200 µL of dd water was added to each thawed fecal sample. The samples were then mixed well by vortexing for 1 min until the materials were homogenized. The pH of the suspension was adjusted to 2–3 by adding 50% sulfuric acid. The acidified samples were kept at room temperature for 5 min and mixed briefly every 60 s. The samples were then centrifuged at 5,000 g for 10 min. The clear supernatant was transferred into two tubes for further processing. 50 µL of the internal standard (1% 2-ethyl butyrate acid solution) and 500 µL of ethyl ether anhydrous were added. The tubes were mixed for 30 s and then centrifuged at 5,000 g for additional 10 min. One µL of the upper ether layer was injected into a gas chromatography instrument Agilent 7820A with a flame ionization detector (Agilent Technologies, CA, USA). A volatile acid mix containing 10 mM of acetic, propionic, butyric, and isovaleric acids was used as an internal standard (Aladdin, Shanghai, China). The retention time and peak heights of the acids in the standard mix were used as references. Individual SCFA were identified by their specific retention time. SCFA concentrations were determined and expressed as mmoles per g of wet feces.

### Fecal total DNA extraction

Fecal samples were collected at necropsy and then snap-frozen in liquid nitrogen and stored at −80 °C until total DNA was extracted. Microbial genomic DNA were extracted from fecal samples with a magnetic bead DNA extraction kit (Sangon, Shanghai, China), according to the manufacturer’s instruction. DNA integrity was verified using a BioAnalyzer 2100 (Agilent, Palo Alto, CA, USA). DNA concentration was then quantified using a QuantiFluor fluorometer (Promega, Madison, WI, USA).

### 16S rRNA gene sequencing

The 16 rRNA gene sequencing was performed as previously described^45^. Briefly, the hypervariable V1-V3 regions of the 16S rRNA gene were directly amplified from 20 ng of total DNA with PAGE-purified Illumina platform-compatible adaptor oligos that contain features such as sequencing primers, sample-specific barcodes, and 16S PCR primers (forward primer, 9F, GAGTTTGATCMTGGCTCAG; reverse primer, 515R: CCGCGGCKGCTGGCAC). The PCR reaction included 2.5 units of AccuPrime TaqDNA Polymerase High Fidelity (Invitrogen, Carlsbad, CA, USA) in a 50 μl reaction buffer containing 200 nM primers, 200 nM dNTP, 60 mM Tris-SO4, 18 mM (NH4)_2_SO4, 2.0 mM MgSO4, 1% glycerol, and 100 ng/uL bovine serum albumin (New England BioLabs, Ipswich, MA, USA). PCR was performed using the following cycling profile: initial denaturing at 95 °C for 2 min followed by 20 cycles of 95 °C 30 s, 60 °C 30 s, and 72 °C 60 s. Amplicons were purified using Agencourt AMPure XP bead kits (Beckman Coulter Genomics, Danvers, MA, USA) and quantified using a BioAnalyzer high-sensitivity DNA chip and a QuantiFluor fluorometer. The purified amplicons from individual samples were pooled in equal molar ratios. The purified amplicon pool was further spiked with approximately 25% of whole-genome shotgun libraries prepared using an Illumina TruSeq DNA sample prep kit with a compatible adaptor barcode to enhance sequence diversity during the first few cycles of sequencing for better cluster differentiation. The concentration of the final library pool was quantified using a BioAnalyzer high-sensitivity DNA chip kit (Agilent). The library pool was sequenced using an Illumina MiSeq Reagent Kit v3 on an Illumina MiSeq sequencer as described previously^40^.

### Sequence data analysis

The sequence data were preprocessed using MiSeq Control Software (MCS) v2.4.1. Raw sequences were first analyzed using FastQC version 0.11.2 to check basic statistics, such as GC%, per base quality score distribution, and sequences flagged as poor quality. The four maximally degenerate bases (“NNNN”) at the most 5′ end of the read pair, which were designed to maximize the diversity during the first four bases of the sequencing run for better identification of unique clusters and improve base-calling accuracy, were then removed. The presence of forward and reverse PCR primers at the 5′ and 3′ ends of each sequence read was scanned; the reads without primers were discarded. Chimeric reads were also removed. The processed pair-end reads were then merged using PandaSeq v2.8 to generate representative complete nucleotide sequences (contigs) using default parameters. The overlapping regions of the pair-end read were first aligned and scored; and reads with low score alignments and high rate of mismatches were discarded.

QIIME pipeline (v1.9.1) was used to analyze the 16S rRNA gene sequences. A “closed reference” protocol in the pipeline was used for OTU picking as previously described^45^. The default QIIME parameters were used, except that the quality-filtering based on OTU abundance threshold was lowered to 0.0001%. GreenGene database (v13.8) was used for taxonomy assignment (greengenes.lbl.gov). PyNAST (v1.2.2) was used for sequence alignment. OTU relative abundance values were then analyzed using the LEfSe algorithm^20^ to identify taxa and KEGG gene families and pathways that display significant differences between two biological conditions. Furthermore, PICRUSt (v1.0.0), a software package designed to predict metagenome functional contents from marker gene surveys, was used with default parameters to predict gene contents and metagenomic functional information based on the OTU table generated using the closed-reference protocol in QIIME. Briefly, the OTU table was first normalized by dividing each OTU by the known/predicted 16S copy number by using the PICRUSt workflow: normalize_by_copy_number.py. The gene contents or the abundance of KEGG Orthology (KO) were predicted from the normalized OTU table using the workflow: predict_metagenomes.py. The predicted metagenome function was further analyzed by collapsing thousands of KEGG Orthologs into higher functional categories (pathways) (categorize_by_function.py). In addition, specific OTU contributing to a given function or pathway was identified by using the work flow: metagenome_contributions.py, as described previously^45^.

## Electronic supplementary material

Dataset 1

## References

[1] Silbert JE, Sugumaran G (2002). Biosynthesis of chondroitin/dermatan sulfate. IUBMB Life.

[2] Mikami T, Kitagawa H (2013). Biosynthesis and function of chondroitin sulfate. Biochim Biophys Acta.

[3] Sato T (2011). Chondroitin sulfate N-acetylgalactosaminyltransferase 1 is necessary for normal endochondral ossification and aggrecan metabolism. J Biol Chem.

[4] Takeuchi K (2013). Chondroitin sulphate N-acetylgalactosaminyl-transferase-1 inhibits recovery from neural injury. Nat Commun.

[5] Kitagawa H, Tsutsumi K, Tone Y, Sugahara K (1997). Developmental regulation of the sulfation profile of chondroitin sulfate chains in the chicken embryo brain. J Biol Chem.

[6] Bergefall K (2005). Chondroitin sulfate characterized by the E-disaccharide unit is a potent inhibitor of herpes simplex virus infectivity and provides the virus binding sites on gro2C cells. J Biol Chem.

[7] Fried M, Duffy PE (1996). Adherence of Plasmodium falciparum to chondroitin sulfate A in the human placenta. Science.

[8] Ayres Pereira M (2016). Placental Sequestration of Plasmodium falciparum Malaria Parasites Is Mediated by the Interaction Between VAR2CSA and Chondroitin Sulfate A on Syndecan-1. PLoS Pathog.

[9] DeAngelis PL (2012). Glycosaminoglycan polysaccharide biosynthesis and production: today and tomorrow. Appl Microbiol Biotechnol.

[10] Ruggiero M, Reinwald H, Pacini S (2016). Is chondroitin sulfate responsible for the biological effects attributed to the GC protein-derived Macrophage Activating Factor (GcMAF)?. Med Hypotheses.

[11] Messier SP (2007). Glucosamine/chondroitin combined with exercise for the treatment of knee osteoarthritis: a preliminary study. Osteoarthritis Cartilage.

[12] Singh JA, Noorbaloochi S, MacDonald R, Maxwell L (2015). J. Chondroitin for osteoarthritis. Cochrane Database Syst Rev.

[13] Barthe L (2004). *In vitro* intestinal degradation and absorption of chondroitin sulfate, a glycosaminoglycan drug. Arzneimittelforschung.

[14] Raghavan V, Lowe EC, Townsend GE, Bolam DN, Groisman EA (2014). Tuning transcription of nutrient utilization genes to catabolic rate promotes growth in a gut bacterium. Mol Microbiol.

[15] Raghavan V, Groisman EA (2015). Species-specific dynamic responses of gut bacteria to a mammalian glycan. J Bacteriol.

[16] Shang Q (2016). Degradation of chondroitin sulfate by the gut microbiota of Chinese individuals. Int J Biol Macromol.

[17] Shang Q (2016). Structural modulation of gut microbiota by chondroitin sulfate and its oligosaccharide. Int J Biol Macromol.

[18] Maes M (2009). Inflammatory and oxidative and nitrosative stress pathways underpinning chronic fatigue, somatization and psychosomatic symptoms. Curr Opin Psychiatry.

[19] Fremont M, Coomans D, Massart S, De Meirleir K (2013). High-throughput 16S rRNA gene sequencing reveals alterations of intestinal microbiota in myalgic encephalomyelitis/chronic fatigue syndrome patients. Anaerobe.

[20] Segata N (2011). Metagenomic biomarker discovery and explanation. Genome Biol.

[21] Langille MG (2013). Predictive functional profiling of microbial communities using 16S rRNA marker gene sequences. Nat Biotechnol.

[22] Clarke SF (2014). Exercise and associated dietary extremes impact on gut microbial diversity. Gut.

[23] Campbell SC (2016). The Effect of Diet and Exercise on Intestinal Integrity and Microbial Diversity in Mice. PLoS One.

[24] Kang SS (2014). Diet and exercise orthogonally alter the gut microbiome and reveal independent associations with anxiety and cognition. Mol Neurodegener.

[25] Evans CC (2014). Exercise prevents weight gain and alters the gut microbiota in a mouse model of high fat diet-induced obesity. PLoS One.

[26] Suchodolski JS (2015). The fecal microbiome in cats with diarrhea. PLoS One.

[27] Shin NR, Whon TW, Bae JW (2015). Proteobacteria: microbial signature of dysbiosis in gut microbiota. Trends Biotechnol.

[28] Suez J (2014). Artificial sweeteners induce glucose intolerance by altering the gut microbiota. Nature.

[29] Carvalho FA (2012). Transient inability to manage proteobacteria promotes chronic gut inflammation in TLR5-deficient mice. Cell Host Microbe.

[30] Powell N (2012). The transcription factor T-bet regulates intestinal inflammation mediated by interleukin-7 receptor + innate lymphoid cells. Immunity.

[31] Rooks MG (2014). Gut microbiome composition and function in experimental colitis during active disease and treatment-induced remission. ISME J.

[32] Li CJ, Li RW (2008). Butyrate induced cell cycle arrest in bovine cells through targeting gene expression relevant to DNA replication apparatus. Gene Regul Syst Bio.

[33] Li RW, Wu S, Baldwin RLt, Li W, Li C (2012). Perturbation dynamics of the rumen microbiota in response to exogenous butyrate. PLoS One.

[34] Campo GM (2009). Glycosaminoglycans modulate inflammation and apoptosis in LPS-treated chondrocytes. J Cell Biochem.

[35] Laparra JM, Lopez-Rubio A, Lagaron JM, Sanz Y (2010). Dietary glycosaminoglycans interfere in bacterial adhesion and gliadin-induced pro-inflammatory response in intestinal epithelial (Caco-2) cells. Int J Biol Macromol.

[36] Miyamoto Y, Itoh K (2000). Bacteroides acidifaciens sp. nov., isolated from the caecum of mice. Int J Syst Evol Microbiol.

[37] Yanagibashi T (2013). IgA production in the large intestine is modulated by a different mechanism than in the small intestine: Bacteroides acidifaciens promotes IgA production in the large intestine by inducing germinal center formation and increasing the number of IgA + B cells. Immunobiology.

[38] Berry D (2013). Host-compound foraging by intestinal microbiota revealed by single-cell stable isotope probing. Proc Natl Acad Sci USA.

[39] Yang, J. Y. *et al*. Gut commensal Bacteroides acidifaciens prevents obesity and improves insulin sensitivity in mice. *Mucosal Immunol*, doi:10.1038/mi.2016.42 (2016). 10.1038/mi.2016.42 27118489

[40] Marques FZ (2017). High-Fiber Diet and Acetate Supplementation Change the Gut Microbiota and Prevent the Development of Hypertension and Heart Failure in Hypertensive Mice. Circulation.

[41] Blasco-Baque V (2017). Associations between hepatic miRNA expression, liver triacylglycerols and gut microbiota during metabolic adaptation to high-fat diet in mice. Diabetologia.

[42] Koropatkin NM, Cameron EA, Martens EC (2012). How glycan metabolism shapes the human gut microbiota. Nat Rev Microbiol.

[43] Dintner S (2011). Coevolution of ABC transporters and two-component regulatory systems as resistance modules against antimicrobial peptides in Firmicutes Bacteria. J Bacteriol.

[44] David LA (2014). Diet rapidly and reproducibly alters the human gut microbiome. Nature.

[45] Li RW (2016). The effect of helminth infection on the microbial composition and structure of the caprine abomasal microbiome. Sci Rep.

